# Supplementary material for: Heterogeneous Folding Intermediates Govern the Conformational Pathway of the RNA Recognition Motif Domain of the Ewing Sarcoma Protein
Source: Biomolecules. 2025 Dec 24;16(1):33. doi: 10.3390/biom16010033 (PMC12839330; doi:10.3390/biom16010033)
Supplement: Supplementary file 1 [file biomolecules-16-00033-s001.zip › biomolecules-4024109-supplementary.pdf]

Supplementary Materials

Heterogeneous Folding Intermediates Govern the Conformational Pathway of the RNA Recognition Motif Domain of the Ewing Sarcoma Protein

Priyanka Kataria [1], Vishakha Chaudhary [1,2], Chandrabhushan Mishra [3], Vijay Kumar [4], Ravi Datta Sharma [5] and Amresh Prakash [1,*]

[1] Data Science Lab., Amity Institute of Integrative Sciences and Health, Amity University Haryana, Gurugram 122413, Haryana, India; piyadhandhi21@gmail.com (P.K.); vishakhabiotech@gmail.com (V.C.)

[2] Amity Institute of Biotechnology, Amity University Haryana, Gurugram 122413, Haryana, India

[3] Department of Pharmacology & Chemical Biology, Baylor College of Medicine, Houston, TX 77030, USA; chandra.medicinalchemist@gmail.com

[4] Amity Institute of Biotechnology, Amity University Noida, Noida 201313, Uttar Pradesh, India;
vkumar33@amity.edu

[5] School of Biotechnology, Shri Mata Vaishno Devi University, Katra 182320, Jammu and Kashmir, India; ravidatta.sharma@smvdu.ac.in

* Correspondence: amreshprakash@jnu.ac.in or aprakash@ggn.amity.edu

Supplementary Figures

Figure S1

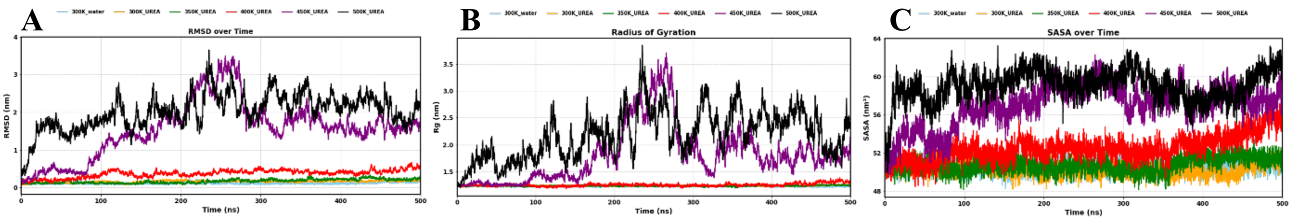

Figure S1 a. *The structural parameter plots: (**A**) Cα- RMSD (**B**) radius of gyration (Rg) and (**C**) SASA of protein, RRM of EWS (PBD ID: 2CPE) during the simulation in Urea at different temperatures (the color code as define above the figure).*

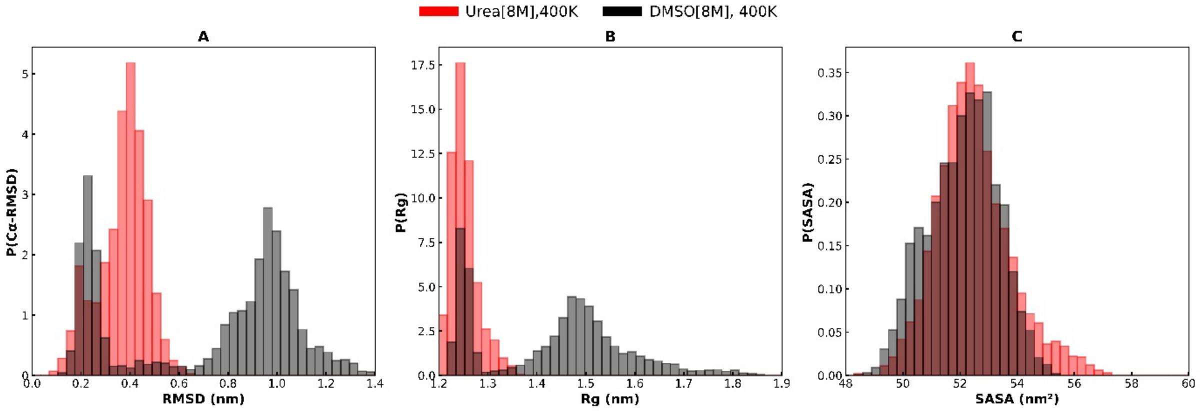

Figure S1 b: *Probability distribution histograms of structural descriptors for the 2CPE protein at 400 K in 8 M urea (red) and 8 M DMSO (black): (A) Cα-RMSD, (B) radius of gyration (Rg), and (C) solvent-accessible surface area (SASA).*

Figure S2

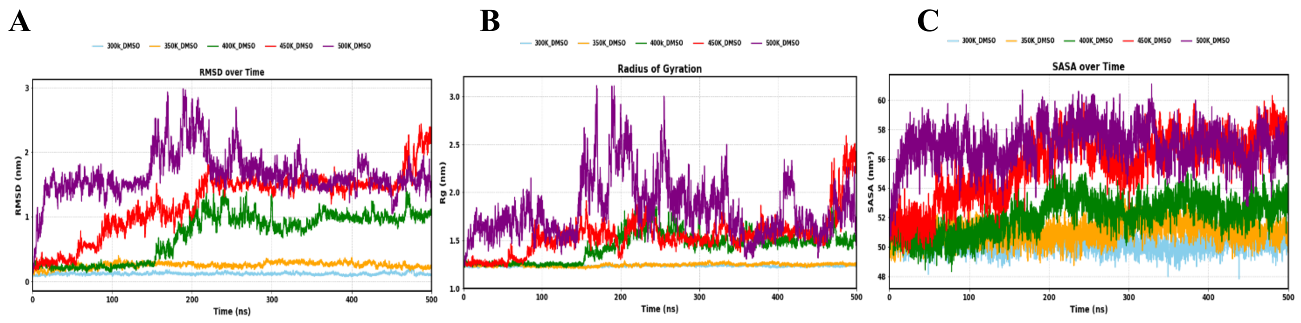

Figure S2a. *The structural parameter plots: (**A**) Cα- RMSD (**B**) radius of gyration (Rg) and (**C**) SASA of protein, RRM of EWS during the simulation in DMSO at different temperatures (the color code as define above).*

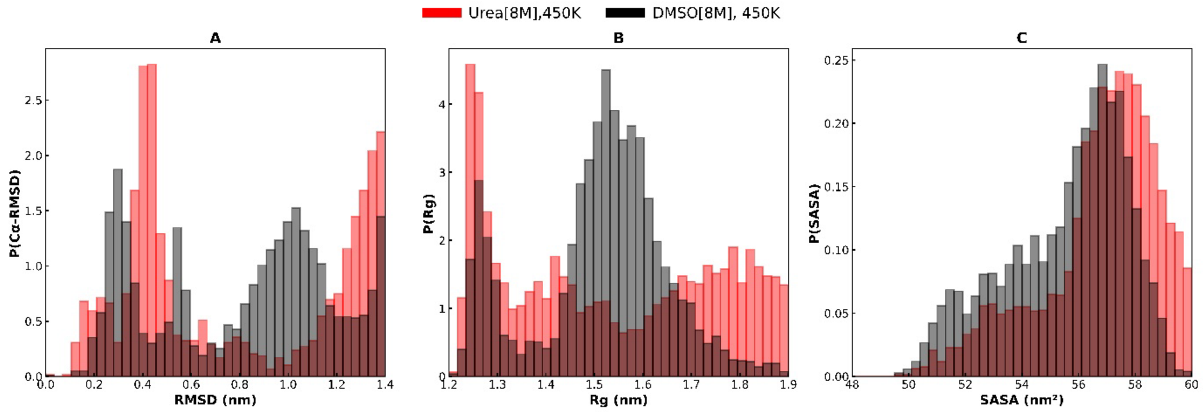

Figure S2b: *Probability distribution histograms of structural descriptors for the 2CPE protein at 450 K in 8 M urea (red) and 8 M DMSO (black): (A) Cα-RMSD, (B) radius of gyration (Rg), and (C) solvent-accessible surface area (SASA).*

Figure S3

(A)

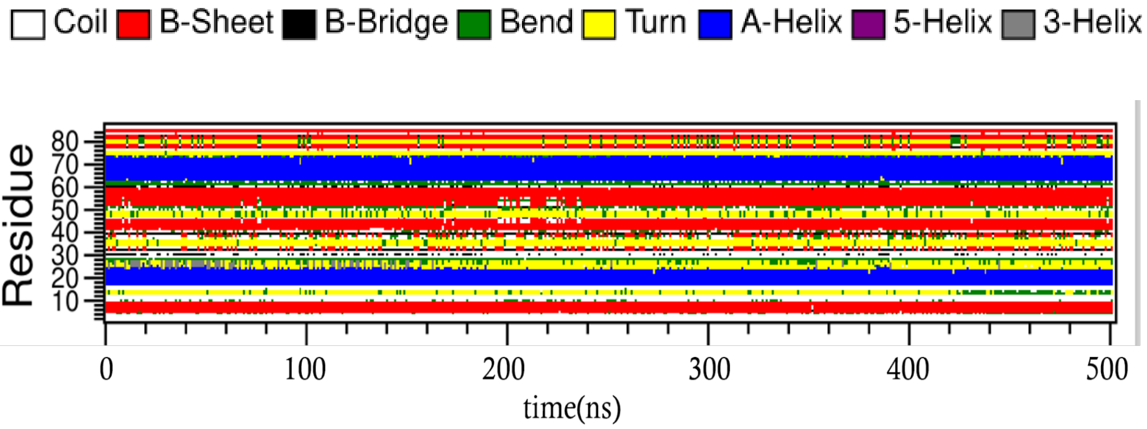

(B)

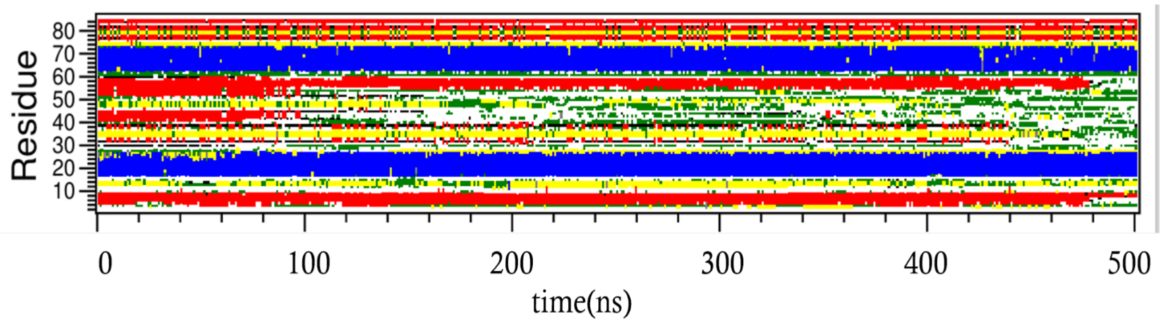

(C)

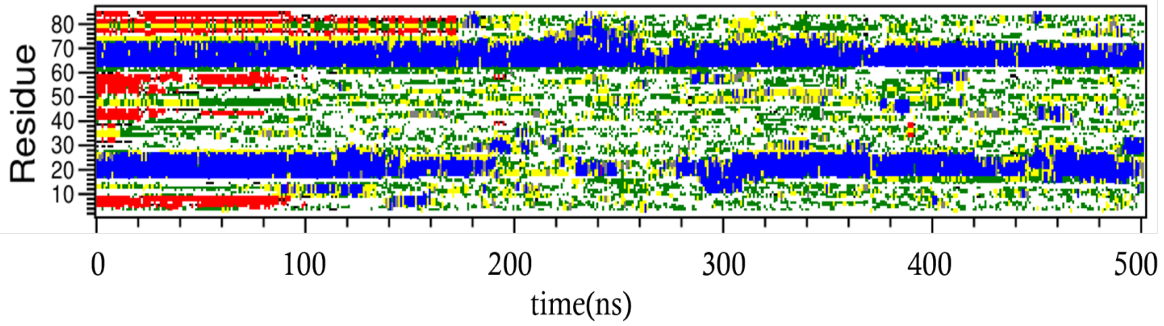

(D)

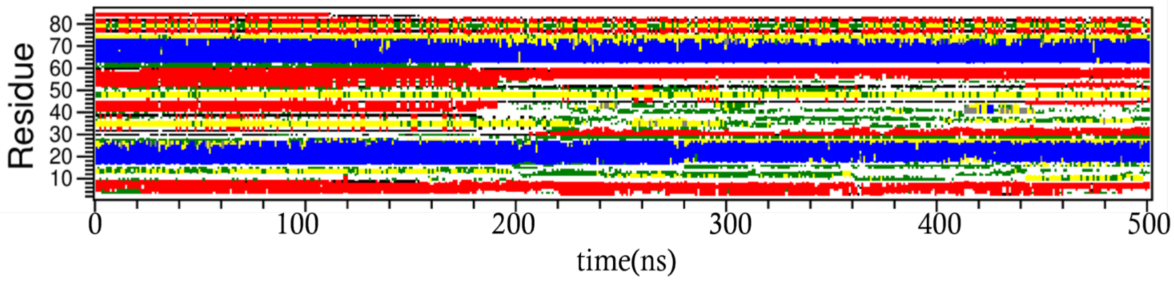

(E)

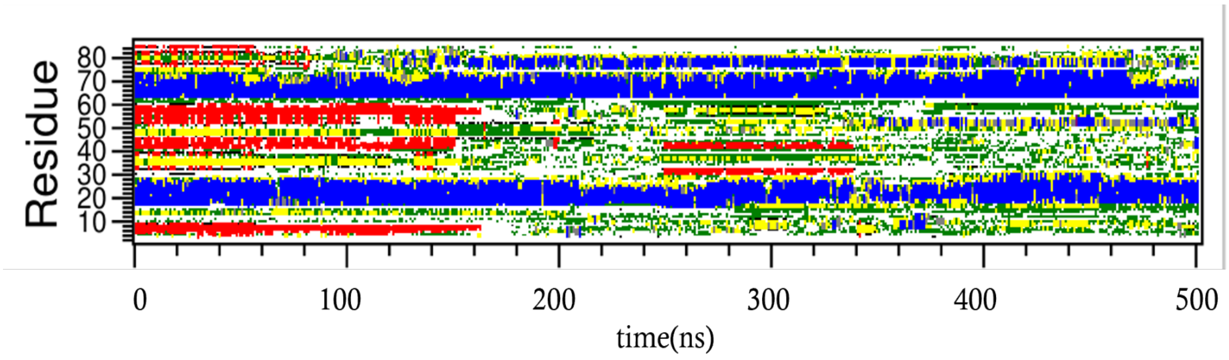

Figure S3: *The time evolution plot of secondary structure of RRM domain of EWS protein. (A) in water, (B) in urea at 400 K (C) in urea at 450 K (D) in DMSO at 400 K and (E) in DMSO at 450 K.*

Figure S4

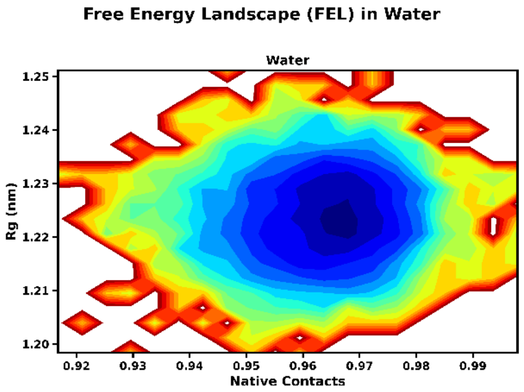

Figure S4. *Free energy landscape plot of protein RRM domain of EWS in water at 300 K. The plot shows the free energy as a function of native contacts (x-axis, Å) and radius of gyration Rg (y-axis, Å),*

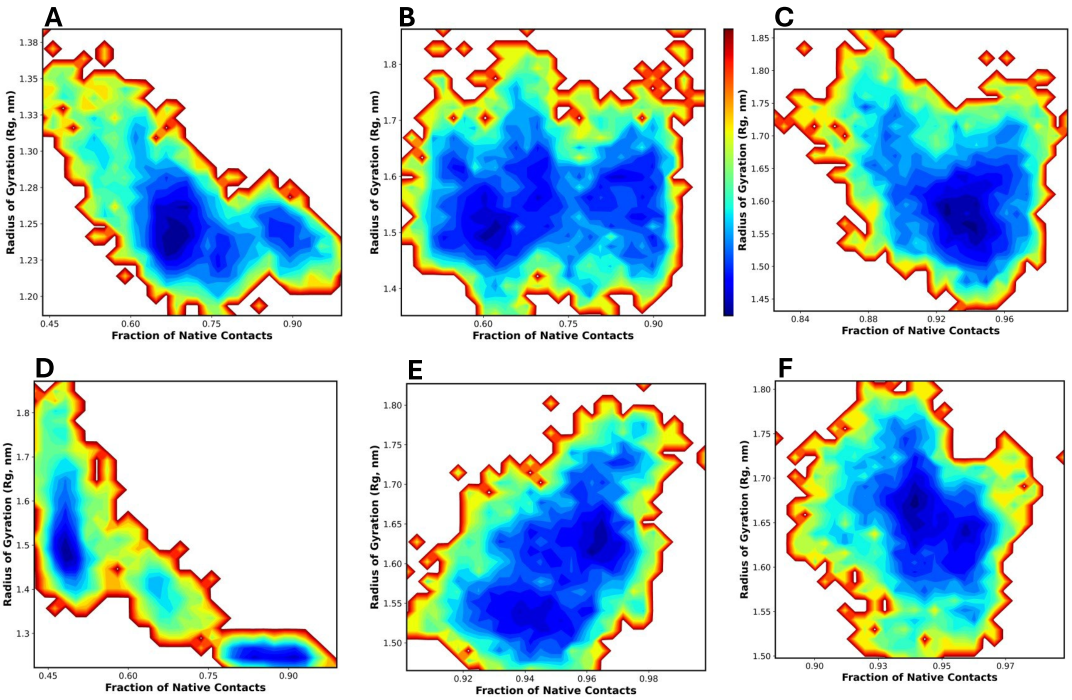

Figure S5. *The FEL contour plots of RRM of EWS (A-C) in urea and (D-F) DMSO at 400 K. The simulations were performed in triplicate, independently in urea and DMSO, respectively.*

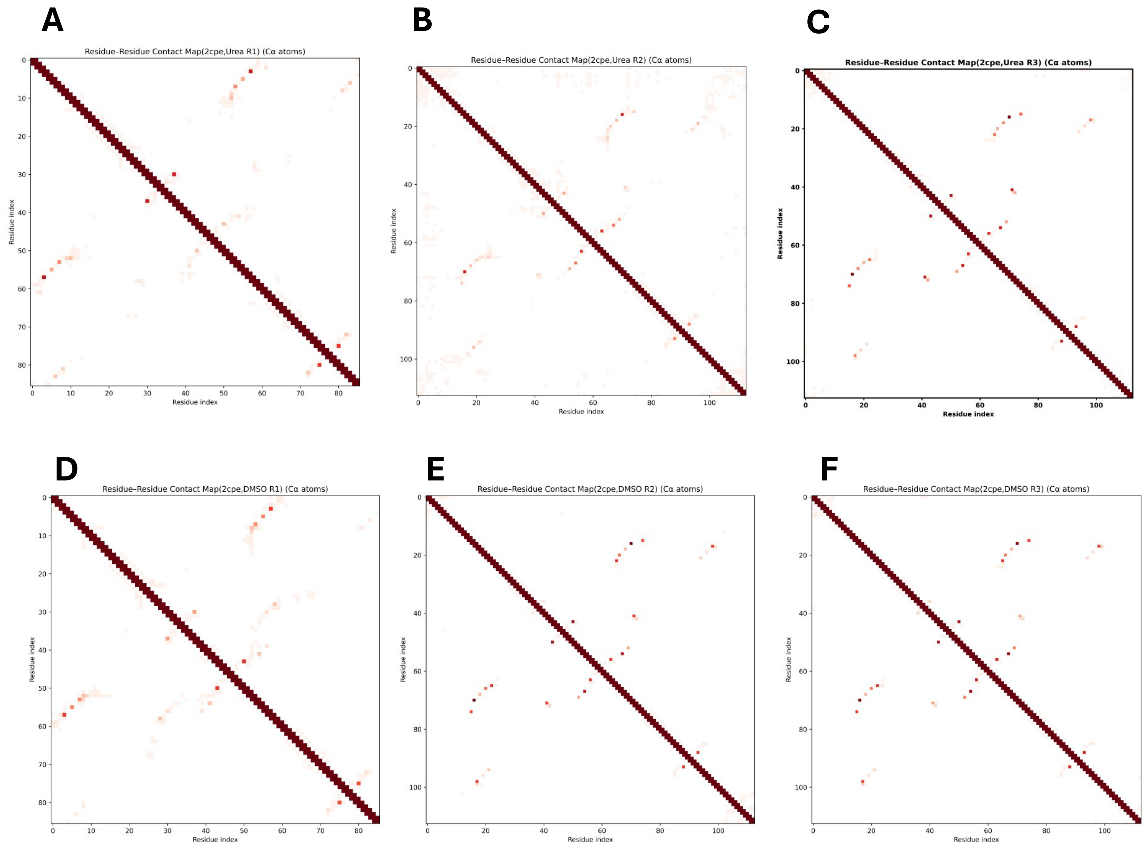

Figure S6. *The backbone (Cα–Cα) contact maps of protein RRM of EWS in urea (A-C) and DMSO (D-F) at 400 K, in triplicate. The contact maps show the pairwise frequency of backbone (Cα–Cα) contacts between residues i (y-axis) and j (x-axis).*

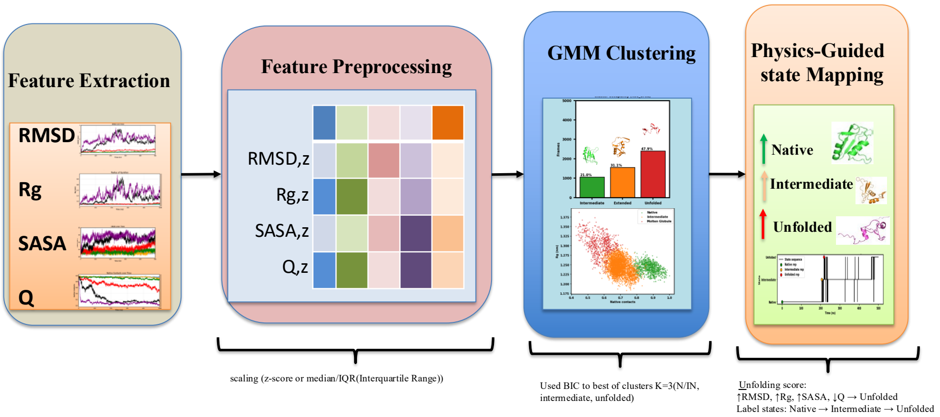

Figure S7. A diagrammatic representation of GMM based clustering of conformational ensemble states.

Details of outlier detection using IQR for GMM analyses:

$$\text{IQR} = Q_3 - Q_1$$
$$\text{Lower } bound = Q_1 - 1.5 \times IQR$$
$$\text{Upper } bound = Q_3 + 1.5 \times IQR$$

Any data point $x$satisfying:

$$x < Q_1 - 1.5\, IQR \; or \; x > Q_3 + 1.5\, IQR$$

was treated as an outlier and excluded prior to clustering.

Robust Feature Scaling

Each observable $x$was scaled as:

$$x_{scaled} = \frac{x - median(x)}{IQR(x)}$$

This ensured comparable weighting of RMSD, Rg, SASA, and Q during clustering.

Gaussian Mixture Model (GMM) Likelihood

The probability density of the GMM is:

$$p(\mathbf{x}) = \sum_{k=1}^{K} \pi_k \; \mathcal{N}(\mathbf{x}, \mid \boldsymbol{\mu}_k \boldsymbol{\Sigma}_k)$$

where $\pi_k$are mixing coefficients, and $\mathcal{N}$represents a multivariate Gaussian distribution.

Bayesian Information Criterion (BIC)

where $\hat{L}$is the maximum likelihood, $p$is the number of model parameters, and $N$is the number of data points. The model with lowest BIC was selected.

Unfolding Score for Physical State Mapping

$$S_{unfold} = \frac{1}{4}\left(Z_{RMSD} + Z_{Rg} + Z_{SASA} - Z_Q\right)$$

Cluster assignment:

with the remaining cluster assigned as Intermediate.
